# Vitamin D and Bone Health; Potential Mechanisms

**DOI:** 10.3390/nu2070693

**Published:** 2010-07-05

**Authors:** Eamon Laird, Mary Ward, Emeir McSorley, J.J. Strain, Julie Wallace

**Affiliations:** School of Biomedical Sciences, University of Ulster, Coleraine, UK; Email: Laird-E1@email.ulster.ac.uk (E.L.); MW.Ward@ulster.ac.uk (M.W.); em.mcsorley@ulster.ac.uk (E.M.); JJ.Strain@ulster.ac.uk (J.J.S.)

**Keywords:** vitamin D, bone, fracture bone mineral density, muscle strength, cytokines

## Abstract

Osteoporosis is associated with increased morbidity, mortality and significant economic and health costs. Vitamin D is a secosteriod hormone essential for calcium absorption and bone mineralization which is positively associated with bone mineral density [BMD]. It is well-established that prolonged and severe vitamin D deficiency leads to rickets in children and osteomalacia in adults. Sub-optimal vitamin D status has been reported in many populations but it is a particular concern in older people; thus there is clearly a need for effective strategies to optimise bone health. A number of recent studies have suggested that the role of vitamin D in preventing fractures may be via its mediating effects on muscle function (a defect in muscle function is one of the classical signs of rickets) and inflammation. Studies have demonstrated that vitamin D supplementation can improve muscle strength which in turn contributes to a decrease in incidence of falls, one of the largest contributors to fracture incidence. Osteoporosis is often considered to be an inflammatory condition and pro-inflammatory cytokines have been associated with increased bone metabolism. The immunoregulatory mechanisms of vitamin D may thus modulate the effect of these cytokines on bone health and subsequent fracture risk. Vitamin D, therefore, may influence fracture risk via a number of different mechanisms.

## 1. Vitamin D Synthesis

For the majority of the population the principle source of vitamin D is synthesis following exposure of the skin to UVB radiation [290-315 nm] [1]. UVB radiation acts in the upper epidermis of the skin with 7-dehydrocholestrol converted to pre-vitamin D3 by photolysis of the B ring structure followed by isomerisation [1]. These structural modifications mean that the molecule no longer conforms within the plasma membrane and is ejected into the extra-cellular space and drawn into the capillary beds where it is bound by the vitamin D binding protein [DBP] and transported to the liver [1]. Maximal production of vitamin D is reached after 10-15 minutes of sun exposure in summer [2] depending on skin pigmentation [3] and during this period one erythaema dose is achieved which is the equivalent to an intake of over 500µg of vitamin D3 [4]. Further UVB exposure results in the formation of in-active photoproducts such as tachysterol and lumisterol that have negligible effects on calcium metabolism [4] and prevent vitamin D toxicity from sun exposure.

Geographical location is a key determinant of the efficiency of vitamin D synthesis as production is dependent on the angle of sunlight or the solar zenith angle [5]. As latitude increases, the amount of light within the wavelength range 290-315 nm to reach the earth’s surface decreases owing to an increased pathway of transversion and ozone absorption. Therefore, at locations above 35° latitude, the synthesis of vitamin D is seasonal with maximal amounts made during summer and little or no synthesis occurring during winter. Synthesis can also be affected by lifestyle, environmental, and physiological factors. Lifestyle factors such as the use of sun-screen, time spent outside and wearing of clothing significantly affect UVB exposure as do environmental factors such as pollution, cloud cover and ozone presence [6,7,8]. For example a sun protective factor [SPF] sunscreen of 15, if correctly applied, would decrease vitamin D synthesis to 1/15th or about 7% [6]. Also a recent cross-sectional study investigating the vitamin D status of 34 children [9-24 months old] living in a polluted area of Delhi reported that pollution reduced vitamin D serum concentrations by 50% [9]. Physiological differences such as skin complexion and age also affect synthesis: darker skin has significantly higher melanin content and subsequently requires a higher UVB exposure time than paler skin to synthesize an equivalent amount of vitamin D [10]. Increasing age is associated with a significant decrease in the amount of 7-dehydrocholesterol in the skin and thus, a reduction in vitamin D synthesis with studies reporting up to four times less cutaneous synthesis in adults aged over 70 years compared to a 20 year old adult [11].

## 2. Dietary Sources

Vitamin D is also obtained to a limited extent from the diet, albeit few dietary sources naturally contain the vitamin in sufficient quantities to make a significant contribution to requirements. The two main vitamin D secosteroids within the diet are vitamin D2 [ergocalciferol] and vitamin D3 [cholecalciferol]. Vitamin D2 is derived from plant and fungi and is produced through the irradiation of ergosterol. Vitamin D3, as previously mentioned, is produced from 7-dehydrocholesterol and is obtained in the diet from animal products with oily fish, fish oils, eggs and dairy produce providing the best dietary sources. A recent double blind, placebo controlled study reported that there is no significant difference in the effectiveness of the two isomers [12]; however it is more often reported that vitamin D3 is the more effective. This higher effectiveness of vitamin D3 over D2 is owing to a possible increased affinity for the vitamin D binding protein [DBP] [13,14] leading to a reduction in clearance of D3 and providing longer lasting concentrations of 25(OH)D in the blood than D2 [14].

In relation to intakes, the composition of the habitual diet will impact on vitamin D status. For example, within Ireland the majority of vitamin D intakes are from sources such as meat and meat products [15] which contain low concentrations of the vitamin. Rich food sources such as oily fish are consumed infrequently. Few vitamin D fortified foods, aside from margarine, are available within Western Europe possibly owing to early adverse advent incidents of infantile hypercalcaemia attributed to over-fortification of dried milk with vitamin D [16]. Other countries, including the USA fortify foods; however, surveys have suggested significant differences between the actual and reported levels of fortification [17,18]. Vitamin D absorption occurs in the ileum and jejunum. It has been estimated that 75% is effectively absorbed [19] but efficiency is dependent on bile salt and micelle formation and, therefore, the presence of malabsorptive disorders such as Coeliac [20] or Crohn’s disease [21] can significantly affect absorption and thus status [22].

## 3. Vitamin D Metabolism and Function [Figure 1]

Vitamin D is bound by the DBP and transported to the liver [1]. Within the liver, the vitamin D is hydroxylated by 25-hydroxylase [CYP2R1] to the major circulating form of the hormone 25-hydroxyvitamin D [25[OH]D3] [23]. Excess non-hydroxylated vitamin D is stored in the liver, adipose tissue and muscle [24]. The 25[OH]D, bound to the DBP, is transported to the proximal tubule cells of kidney. The DBP is degraded and the 25[OH]D3 is hydroxylated by 1α-hydroxylase [CYP27B1] to the biologically active form of the hormone, 1,25 di-hydroxyvitamin D3 [1,25[OH]2D3] [calcitriol] [25]. The kidney however, is not the only source of CYP27B1: it has also been detected in tissues such as the colon, brain and pancreas suggesting different autocrine functions of the hormone [25]. Regulation of CYP27B1 within the renal tubules is controlled by fibroblast growth factor [FGF-23] and by parathyroid hormone [PTH]. PTH stimulates while FGF-23 inhibits 1α-hydroxylase production in the kidney through a series of feedbacks [23,24]. Once the hormone has been metabolized it is converted to calcitroic acid and excreted. The primary effect of vitamin D is enhanced calcium absorption in the small intestine [23,24,25]. With hypocalcaemia, increased PTH secretion stimulates production of 1,25[OH]2 D3 in the kidney [25]. The hormone interacts with the vitamin D receptor [VDR] in intestinal cells and complexes with the retinoic acid x receptor [RXR] in the nucleus [23,24]. This complex binds to the vitamin-D-responsive element [VDRE] of the calcium channel [TRPV6] which increases uptake of calcium into the cells and increases the absorption of calcium [24,25]. Secondly, within osteoblasts, vitamin D interacts with the VDR and increases the plasma membrane expression of the RANKL [Receptor Activator for Nuclear Factor κ B Ligand] [25]. RANK on preosteoclasts binds RANKL on the osteoblast which then converts the preosteoclast to an osteoclast [25]. This conversion releases chemicals such as hydrochloric acid to metabolise calcium stores from the bones into circulation to maintain the optimal physiological range [25]. 

**Figure 1:**
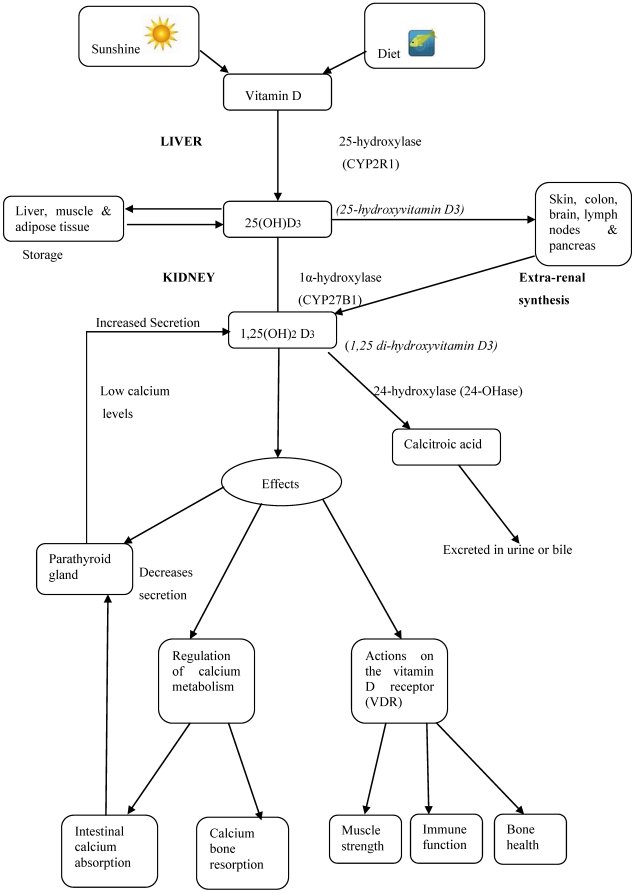
Summary diagram of vitamin D metabolism and function.

## 4. Vitamin D Intake and Status

Until recently, it was generally assumed that enough vitamin D was synthesized from sun exposure to meet requirements and no recommendations for dietary intakes for adults aged 18-65 yrs were set. However, research highlighting the effects of low vitamin D status [26,27] has indicated the importance of vitamin D in the diet. Within Ireland, the Food Safety Authority for Ireland [FSAI] has set a wide range of 0-10 μg/d (day) for adults [28] while in the UK there is no reference nutrient intake [RNI] for adults [18-64 yrs], only for those over 65 yrs [10 μg/d] and pregnant and lactating women [10 μg/d] [29]. In the US, adequate intakes [AI] are set at 5 μg/d [19-50 yrs] and 10 μg/d [51-70 yrs] [30] which also correspond with the WHO/FAO Expert Group recommended intakes [31]. 

The difficulty in assigning a RNI for vitamin D intake is exacerbated by the current lack of agreement on optimal vitamin D status. While vitamin D deficiency is generally accepted as a 25[OH]D3 serum concentration <25 nmol/L [32], the serum concentrations of 25[OH]D3 defining insufficiency, or optimal status are less clear. Insufficiency can be deemed as mild deficiency with a 25[OH]D3 serum concentration <50 nmol/L [33,34,35]. Over extended periods of time, insufficiency has been associated with increased bone loss and secondary hyperparathyroidism leading to increased fracture risk [36]. Sufficiency has been regarded as the point at which further intakes will have no additional beneficial effects on PTH and calcium metabolism in regard to bone health. However, the cut-off values for sufficiency are still under debate. Studies have demonstrated concentrations of >80 nmol/L [37] or higher, 100-200 nmol/L [35], as optimal. 

As mentioned earlier, the dietary intake of vitamin D required to prevent vitamin D deficiency and ensure optimal vitamin D status will vary depending on sun exposure preferences. A recent study concluded that to maintain 25[OH]D >25 nmol/L in 97.5% of the population during wintertime the recommended dietary allowances [RDA] should be set at 8.7ug/d. However, the authors also calculated that a dietary intake of 12.1ug/d would be required to prevent deficiency among sun avoiders [38]. Such intakes are considerably higher than estimated vitamin D intakes in Ireland [3.3 µg] [39], the UK [4.2 µg] [40], the US [8.12 µg] [41] and other European countries [42,43,44]. 

Vitamin D toxicity is rare [45]. The symptoms include vomiting, nausea, constipation, weight loss, weakness and kidney stones with subsequent hypercalcaemia and ectopic calcification of soft tissue [46]. However, as discussed earlier, the formation of vitamin D from sunlight is a self-limiting reaction; thus preventing toxicity from sun exposure. Reports of toxicity have arisen from excessive dietary intakes of the vitamin [17,47,48,49] with all such cases reporting serum 25[OH]D concentrations >200 nmol/L. However, severe adverse effects have only been reported with extreme intakes of vitamin D of 15,000 µg/d [47,48] or 42,000 µg/d [49], well above any natural intakes from food or supplements from clinical trials. 

## 5. Vitamin D and Bone Health

Peak bone mass is attained by the third decade of life [50] with genetics, physical activity, nutrition and lifestyle factors [51,52] playing key roles in the accumulation and maintenance of bone. Age related bone loss occurs around the fourth decade [53], resulting in a gradual decline of BMD though this process is accelerated in females during and up to 10 years post-menopause owing to possible oestrogen deficiency derived bone loss [54]. The development of bone disease in later life is related to the attainment of maximum peak bone mass and the maintenance of bone mass in adulthood [55]. In relation to vitamin D, research has shown that inadequate vitamin D intakes over long periods of time can lead to bone demineralization [56]. Vitamin D deficiency leads to decreased calcium absorption and ultimately the release of calcium from the bones in order to maintain circulating calcium concentrations [56]. Continuous bone turnover and resorption weakens the architecture of bones and increases fracture risk via secondary hyperparathyroidism [56] ultimately leading to the development of osteomalacia and osteoporosis. Osteoporosis is clinically defined as a BMD 2.5 standard deviations below the mean of healthy young individuals [57,58].

There is a direct relationship between BMD and fracture risk [59], with a decrease in bone strength and density associated with an increased incidence rate of fractures [60].Fractures usually occur at the hip, spine and wrist [61] and not only carry considerable health costs, but can also result in increased mortality and a decreased quality of life [62,63,64]. Fracture incidence has been shown to increase with age [65]; therefore it has been imperative to develop preventive strategies in order to minimize the development of this condition. Given the relationship between vitamin D and bone mineralization, optimal vitamin D status is essential for minimization of fracture risk.

## 6. Fracture Risk

A number of studies have investigated the effects of vitamin D or vitamin D in combination with calcium on fracture incidence (Table 1,Table 2,Table 3). Three intervention trials of vitamin D alone [66,75,77] have reported a significant reduction in fracture occurrence. For example, one study [75] reported a 33% lower rate for fracture and a 22% lower rate for first fracture at any site compared to placebo in free-living individuals with 100,000 IU D3. While in another intervention [66], which supplemented with 150,000-300,000 IU D2, an apparent significant fracture reduction from baseline was reported, especially in the upper limb both in free-living and institutionalized subjects [though this study had no placebo and was not blinded]. Furthermore, in a trial [77] supplementing post-stroke elderly patients with 1000 IU D2, zero hip fractures were reported in the vitamin D arm (n = 48). These results are also supported by trials with vitamin D in combination with calcium. For example, in one study [89], supplementation with 400 IU D3 and calcium resulted in a reduction in the fracture incidence rate of community dwelling individuals. Furthermore, another trial [81] reported a significant reduction in hip fractures among institutionalized elderly females supplemented with calcium and vitamin D3 for two years. A subsequent analysis of the same cohort after 42 months supplementation indicated that hip fractures and non-vertebral fractures remained lower in the treatment group [94]. However, not all trials of vitamin D or vitamin D in combination with calcium have reported significant effects. For example, Lyons *et al.* [79] in a double-blinded, placebo-controlled trial, supplemented nursing home residents with 100,000 I.U D2 quarterly for five years and reported no significant reduction in fractures. Moreover, in a much larger trial [92] of 36,000 subjects with 400 IU D3 & 500 mg calciumno significant reduction in fractures was reported. The difference in outcomes between trials is difficult to explain, albeit there are a number of possible explanations for lack of reported effects. A large number of trials [69,71,74,76,85,87,88,91,92,93] have supplemented with between 300-800 IU vitamin D which may not be enough to exert a beneficial effect according to a recent meta-analysis [95,96]. In addition, studies which have documented significant fracture reductions have reported that effects may be more evident among institutionalized elderly people compared to free-living individuals. 

These observations are supported by a number of meta-analyses. Bischoff-Ferrari (2005) conducted a meta-analysis [97] of 7 RCTs and concluded vitamin D in the range of 700-800 IU/d reduced the risk of hip/non-vertebral fractures by 25% while a calcium intake of more than 700 mg/d was required for non-vertebral fracture prevention. In a separate analysis [98], an 18% reduction in hip fracture risk and a significant reduction in non-vertebral fractures were achieved with vitamin D and calcium supplementation. It has also been reported that vitamin D and calcium intervention is more effective in reducing the risk of hip fracture in patients in institutionalized care compared to free living individuals in the community [94,95] with more than 800 IU vitamin D and more than 1200 mg calcium daily required respectively to exert a beneficial effect. In keeping with this finding of a threshold effect of vitamin D, a more recent analysis [99] concluded that supplementation with 400 IU/d or less was ineffective while doses between 482-770 IU/d reduced fractures [hip 18%, non-vertebral 20%] with no further effect with additional calcium supplementation. Conversely, a recent Cochrane analysis concluded that vitamin D alone did not have a significant effect on fracture prevention and was only effective when combined with calcium in institutionalized individuals [100]. This finding is supported by a recent pooled analysis of over 68,000 patients which reported that 400-800 IU/d of vitamin D alone was not effective but 400 IU/d combined with calcium reduced the rate of hip fracture by 16% and reduced overall fractures by 8% [101]. Two meta-analyses to date [99,102] have looked at the effect of vitamin D analogues with one concluding that calcitriol & alfacalcidol were more effective at reducing fracture risk than D2 or D3 [102]. The use of such analogues, however, is not widely advocated at present given the potential risks and cost implications associated with them [99].

Taken together, the results from the meta-analyses support a cause and effect relationship between vitamin D in combination with calcium in the reduction of risk of vertebral and non-vertebral osteoporotic fractures. Several mechanisms may underlie this positive association between vitamin D and fracture risk including the well documented beneficial effects of vitamin D on bone mineral density.

**Table 1:** Intervention with vitamin D on fracture prevention.

Study	Study design	n	Sex	Mean age [years]	Treatment	Length	Results
Heikinheimo *et al.* 1992 (66)	CT	320	M/F	75-84	150,000-300,000 IUD2 yrly	5 yrs	Groups with vitamin D had sig lower rate of fractures,
		479	M/F	>85	150,000-300,000 IU D2 yrly	5 yrs	particularly upper limb but not lower limb fractures
Lips *et al.* 1996 (69)	CT	2578	M/F	80	400 IU D3/d	3.5 yrs	No effect
Peacock *et al*. 2000 (71)	CT	438	M/F	74	800 IU D3/d	4 yrs	No effect
Meyer *et al*. 2002 (74)	CT	1144	M/F	84	400 IU D3/d	2 yrs	No effect
Trivedi *et al*. 2003 (75)	CT	2686	M/F	75	100 000 IU D3 four monthly	5 yrs	33% lower rate for fracture at hip, forearm and vertebrae
Grant *et al.* 2005 (76)	CT	2675	M/F	77	800 IU D3/d	2 yrs	No effect
Sato *et al*. 2005(77)	CT	96	F	74	1, 000 IU D2/d	2 yrs	Reduction in hip fracture
Law *et al*. 2006 (78)	OSD	3717	M/F	85	1,100 IU D2/d	10 mths	No effect
Lyons *et al*. 2006 (79)	CT	3440	M/F	84	100 000 IU D2 four monthly	3 yrs	No effect
Smith *et al*. 2007 (80)	CT	9440	M/F	79	300 000 IU D2/d	3 yrs	No effect
CT, controlled trial;OSD, open study design trial; F, female; M, male; D3, cholecalciferol; D2, ergocalciferol; Ca, calcium;/d, daily; mths, months; yrly, yearly; IU, International units							

**Table 2:** Interventionwith vitamin D plus calcium on fracture prevention.

Study	Study design	n	Sex	Mean age [years]	Treatment	Length	Results
Chapuy *et al.* 1992 (81)	CT	3270	F	84	800 IU D3 & 1200 mg Ca/d	2 yrs	Hip fractures 43% & non-vertebral fractures 32% lower
Dawson Hughes *et al*. 1997 (84)	CT	389	M/F	71	700 IU D3 & 500 mg Ca/d	3 yrs	Reduction in non-vertebral fractures
Komulainen *et al*. 1999 (85)	CT	464	F	52	300 IU D3 (100 IU in last yr) & 93 mg Ca/d	5 yrs	Noeffect
Chapuy *et al*. 2002 (87)	CT	583	F	85	800 IU D3 & 1200 mg Ca/d	2 yrs	No effect
Harwood *et al*. 2004 (88)	OSD	76	F	82	800 IU D3 & 1000 mg Ca/d	1 yr	No effect
Larsen *et al*. 2004 (89)	CT	9605	M/F	74	400 IU D3 & 1000 mg Ca/d	3 yrs	16% reduction in fracture risk
Flicker *et al*. 2005 (90)	CT	625	M/F	83	10,000 IU D2 wk to 1000 IU/d& 600 mg Ca/d	2 yrs	No effect
Porthouse *et al*. 2005 (91)	OSD	3314	F	70	800 IU D3 & 1000 mg Ca/d	25 mths	No effect
Grant *et al.* 2005 (76)	CT	2638	M/F	77	800 IU D3 & 1000 mg Ca/d	2 yrs	No effect
Jackson *et al*. 2006 (92)	CT	36282	F	62	400 IU D3 & 500 mg Ca/d	7 yrs	No effect
Pfeifer *et al*. 2009 (93)	CT	242	M/F	77	800 IU D3/1000 mg Ca/d	2 yrs	No effect
CT, controlled trial;OSD, open study design trial; F, female; M, male; D3, cholecalciferol; D2, ergocalciferol; Ca, calcium;/d, daily; yr, year; yrs, years							

**Table 3:** Intervention with vitamin D analogues (with / without calcium) on fracture incidence.

Study	Study design	n	Sex	Mean age (years)	Treatment	Length	Results
**Vitamin D analogues **							
Hayashi *et al*. 1992 (67)	CT	740	M/F	75	40 IU Ac/d	1 yr	Lower fracture incidence
Tyliard *et al*. 1992 (68)	CT	622	F	63	20 IU Cl/d	3 yrs	Reduction in vertebral fractures
Sato *et al*. 1999 (70)	CT	86	M/F	70	40 IU Ac/d	2 yrs	17.5% lower fracture rate
Ebeling *et al*. 2001 (72)	CT	41	M	57.5	20 IU Cl/d	2 yrs	No effect
Gallagher *et al*. 2001 (73)	CT	489	F	72	20 IU Cl/d	2 yrs	No effect
**Vitamin D analogues with calcium**							
Orimo *et al*. 1994 (82)	CT	80	F	70	40 IU Ac & 300mg Ca/d	1 yr	Decreased vertebral fracture incidence
Shikari *et al*. 1996 (83)	CT	113	F	70	30 IU Ac & 300mg Ca/d	2 yrs	Decreased incidence of fractures
Stempfle *et al*. 1999 (86)	CT	132	M/F	50	10 IU Cl & 1000mg Ca/d	3 yrs	No effect
CT, controlled trial; F, female; M, male; Cl, Calcitriol; Ac, Alfacalcidol; Ca, Calcium; /d, daily; wkly, weekly; yr, year;yrs, years							

## 7. Vitamin D and BMD

A number of cross-sectional studies have investigated the relationship between vitamin D status and BMD. For example, one study [103] of middle aged women reported that serum concentrations of 25[OH]D were positively related to bone density of the lumbar spine, neck and trochanteric regions of the femur. In addition, a more recent study [104] reported a positive association of vitamin D and BMD and suggested the effect may be even greater in the 90-100 nmol/L serum range of vitamin D. Given the observed relationship between vitamin D status and BMD, a large number of intervention studies have investigated the effects of vitamin D alone or vitamin D in combination with calcium on BMD (Table 4,Table 5, Table 6).

Vitamin D alone and in combination with calcium appears to have a clear significant effect on BMD. For example, 5 out of 9 studies of vitamin D alone [70,73,105,106,107], plus 16 of 22 studies [81,84,88,92,94,98,110,111,112,116,117,118,120,122] of vitamin D in combination with calcium have reported significant positive effects on BMD. For example, in supplementation with vitamin D alone, one study [107] reported a significant benefit after only five weeks in participants with impaired vitamin D status and osteoporosis/osteopenia. Supplementation with 100 000 IU D2 resulted in rapid improvement in vitamin D status and significant improvement in spinal and the femoral neck BMD. The findings are supported by another trial [106] of over 300 elderly mobile females in whom two years of vitamin D3 supplementation resulted in significant improvements in BMD. The improvements in BMD can also be seen in the trials with vitamin D and calcium. Over the last 20 years, 14 intervention studies examining the effect of vitamin D and calcium supplementation on BMD have been published. Overall, positive results have been reported, with doses more than 400 IU associated with significant effects on bone heath. However, the optimal dosage of vitamin D remains unclear. For example, two trials [117,120] which supplemented with 560 IU D3 & 1000 mg calcium/d [117] or 560 IU D3 and 500 mg Ca/d [120] reported increases in lumbar spine BMD while conversely; another trial [115] supplementing with 500 IU D3 and 1000 mg calcium/d over the same intervention period reported no significant effect. Four trials have reported significant increases in BMD with 700 IU [84,112] to 800 IU [81,88] D3 and calcium [500-1,200 mg]; however, in a study that supplemented African-American females with 800 IU D3 and 1,200-1,500mg of calcium [121] no significant effects were reported. The findings in African-American females could be explained, in part, by lower bone remodelling rates [124] and enhanced skeletal resistance to the effects of PTH [125] in this population group. Studies intervening with higher doses of vitamin D have also produced conflicting results.For example, one trial [120], supplementing with 1428 IU D2 and 1000 mg calcium/d reported no effect while another [123] supplementing 5000 IU D3/d with 320mg calcium reported significant effects. In the latter trial, 45 elderly participants were supplemented with vitamin D3 through bread fortification. An impressive 4% increase in spine and 23.4% in hip BMD were reported. Trials (Table 5) supplementing with vitamin D analogues have also reported significant improvements in BMD. For example, in one trial [73], 20 IU alone resulted in a significant increase in spine BMD. Trials [98,99,111,116,118,119] with vitamin D analogues and calcium have also reported consistent results with the majority reporting significant improvements in BMD [70,73,98,99,111,116,118,119]. 

Vitamin D alone and in combination with calcium appears to have a clear significant effect on BMD. For example, 5 out of 9 studies of vitamin D alone [70,73,105,106,107], plus 16 of 22 studies [81,84,88,92,94,98,110,111,112,116,117,118,120,122] of vitamin D in combination with calcium have reported significant positive effects on BMD. For example, in supplementation with vitamin D alone, one study [107] reported a significant benefit after only five weeks in participants with impaired vitamin D status and osteoporosis/osteopenia. Supplementation with 100 000 IU D2 resulted in rapid improvement in vitamin D status and significant improvement in spinal and the femoral neck BMD. The findings are supported by another trial [106] of over 300 elderly mobile females in whom two years of vitamin D3 supplementation resulted in significant improvements in BMD. The improvements in BMD can also be seen in the trials with vitamin D and calcium. Over the last 20 years, 14 intervention studies examining the effect of vitamin D and calcium supplementation on BMD have been published. Overall, positive results have been reported, with doses more than 400 IU associated with significant effects on bone heath. However, the optimal dosage of vitamin D remains unclear. For example, two trials [117,120] which supplemented with 560 IU D3 & 1000 mg calcium/d [117] or 560 IU D3 and 500 mg Ca/d [120] reported increases in lumbar spine BMD while conversely; another trial [115] supplementing with 500 IU D3 and 1000 mg calcium/d over the same intervention period reported no significant effect. Four trials have reported significant increases in BMD with 700 IU [84,112] to 800 IU [81,88] D3 and calcium [500-1,200 mg]; however, in a study that supplemented African-American females with 800 IU D3 and 1,200-1,500mg of calcium [121] no significant effects were reported. The findings in African-American females could be explained, in part, by lower bone remodelling rates [124] and enhanced skeletal resistance to the effects of PTH [125] in this population group. Studies intervening with higher doses of vitamin D have also produced conflicting results.For example, one trial [120], supplementing with 1428 IU D2 and 1000 mg calcium/d reported no effect while another [123] supplementing 5000 IU D3/d with 320mg calcium reported significant effects. In the latter trial, 45 elderly participants were supplemented with vitamin D3 through bread fortification. An impressive 4% increase in spine and 23.4% in hip BMD were reported. Trials (Table 5) supplementing with vitamin D analogues have also reported significant improvements in BMD. For example, in one trial [73], 20 IU alone resulted in a significant increase in spine BMD. Trials [98,99,111,116,118,119] with vitamin D analogues and calcium have also reported consistent results with the majority reporting significant improvements in BMD [70,73,98,99,111,116,118,119]. 

In summary, the majority of vitamin D supplementation trials demonstrate a positive effect of the vitamin on BMD and in de facto fracture incidence although further studies are required to confirm the optimal dose of vitamin D associated with these benefits. Moreover, although the potential benefits of vitamin D on fracture incidence may be at least partly attributed to beneficial effects on BMD, vitamin D may also impart advantages by reducing fracture occurrence through other mechanisms. One such mechanism is based on the potential of vitamin D to enhance muscle strength and thereby decrease the risk of falls.

## 8. Vitamin D and Muscle Strength

A recent review [126] concluded that falls were the biggest contributor to fractures and observational studies have reported that vitamin D deficiency is associated with muscle weakness [127] and an increased pre-disposition for falling [128]. Such associations are not surprising given that research has shown that the VDR is expressed in both skeletal muscle [129] and myoblast cells [130]. *In vitro* evidence has demonstrated that vitamin D can increase protein synthesis and cellular growth in muscle cells with an increase in size and number of type 2 muscle fibres [131] which are of particular importance as these are the first muscle fibres recruited when falling [132]. In support of these findings, a recent observational study [133] has reported vitamin D plasma concentrations in the range of 40-90nmol/L was associated with improved musculoskeletal function than serum concentrations <40nmol/L. Additionally, another study [134] has suggested that impaired muscle function in vitamin D deficient subjects could be present even before indications of bone disease have been recognised. Such evidence clearly provides a mechanism to support an improvement in muscle strength and in defacto bone health following vitamin D supplementation.

A number of intervention trials have investigated the effects of vitamin D supplementation on muscle with contrasting results. One of the earliest supplementation studies reported that 40 IU alfacalcidol administered over a period of between 3 and 6 months increased the size and number of type 2 muscle fibres [131]; however, a subsequent study using a lower dose [20 IU alfacalcidol] for three years reported no effect of supplementation on muscle strength [135]. In the later study, baseline vitamin D status was reported at 60nmol/L, potentially reducing any likely beneficial effect of supplementation. Furthermore, a single 300,000 IU dose of vitamin D3 failed to exert a beneficial effect in frail elderly individuals, even in those deficient in vitamin D [136]. In contrast, Sato *et al*. [77] reported that daily supplementation with 1000 IU vitamin D2 for two years produced a significant increase in type 2 muscle fibre diameter in hospitalized elderly females with post-stroke hemiplegia. More recently, two studies have reported significant associations with vitamin D and muscle strength [137,138] with one study reporting a significant association between vitamin D and lower extremity function. The observed beneficial effects of vitamin D on muscle could be explained by its modulating effects on PTH as hyperparathyroidism has been reported to induce muscle weakness and atrophy of type 2 muscle fibres in animal models [139]. The observations, in turn, could be explained by the promoting effects of PTH on pro-inflammatory cytokines [140], which are often considered co-factors for muscle wasting and disability, especially in older individuals [141]. For example, PTH has been shown to increase production of IL-6 *in vivo* [140], which has been linked with muscle weakness and bone resorption.

**Table 4:** Intervention with vitamin D (with / without calcium) on bone mineral density (BMD).

Study	Study design	n	Sex	Mean age (years)	Treatment	Length	Results
**Vitamin D **							
Nordin *et al*. 1985 (105)	CT	109	F	65-74	15,000 IU D2 wkly	2 yrs	Reduced rate of metacarpal cortical bone loss
Ooms *et al*. 1995 (106)	CT	348	F	80	400 IU D3/d	2 yrs	Inc. in BMD; femoral neck; 1.9% in left, 2.6% in right
Adams *et al.* 1999 (107)	IT	12	F	60	100 000 IU D2/wkly	5 weeks	Inc. in BMD; femoral neck (4.9%) & spine (4.1%)
Hunter *et al*. 2000 (108)	CT	128	F	58.7	800 IU D3/d	2 yrs	No effect
Peacock *et al*. 2000 (71)	CT	438	M/F	74	800 IU D3/d	4 yrs	No effect
Patel *et al*. 2001(109)	CT	70	F	47.2	800 IU D3/d	2 yrs	No effect
**Vitamin D with calcium**							
Dawson Hughes *et al*. 1991(110)	CT	249	F	81	400 IU D3 & 377 mg Ca/d	1 yr	Reduced winter time bone loss/improved BMD of spine
Chapuy *et al.* 1992 (81)	CT	3270	F	84	800 IU D3 & 1200 mg Ca/d	2 yrs	BMD of femur increased by 2.7% compared to placebo
Dawson Hughes *et al*. 1995 (112)	CT	247	F	63	700 IU D3 & 500 mg Ca/d	3 yrs	1.5% inc. in BMD in femoral neck in 700 IU group only
Adachi *et al*. 1995 (113)	CT	62	M/F	64	7142 IU D3 & 1000 mg Ca/d	35mths	No effect
Bernstein *et al*. 1996 (114)	CT	24	M/F	35	250 IU D3 & 1000 mg Ca/d	1yr	No effect
Buckley *et al*. 1996 (115)	CT	66	M/F	52	500 IU D3 & 1000 mg Ca/d	2 yrs	No effect
DawsonHughes *et al*. 1997 (84)	CT	389	M/F	71	700 IU D3 & 500 mg Ca/d	3 yrs	Inc. in BMD
Baeksgaard *et al*. 1998(117)	CT	240	F	62.5	560 IU D3 & 1000 mg Ca/d	2 yrs	1.6% inc. in lumbar spine BMD
Komulainen *et al*. 1999 (85)	RT	464	F	52	300 IU C/c/d (100IU D3/d 5th year) & 93 mg Ca/d	5 yrs	No effect
Cooper *et al*. 2003(120)	CT	187	F	56	1,428 IU D2 & 1000 mg Ca/d	2 yrs	No effect
Meier *et al*. 2004 (121)	OSD	55	M/F	56	500 IU D3 & 500 mg Ca/d	2 yrs	0.8 % inc. in lumbar spine BMD
Harwood *et al*. 2004 (88)	OSD	76	F	82	800IU D3 & 1000 mg Ca/d	1 yr	Change in Hip BMD
Aloia *et al*. 2005 (122)	CT	280	F	50-75	800 IU D3/d (2000IU after 2yrs)	3 yrs	No effect
Jackson *et al*. 2006 (92)	CT	36282	F	62	400 IU D3 & 500 mg Ca/d	7 yrs	Change in Hip BMD (+1.06% compared to placebo)
Mocanu *et al*. 2009 (123)	SAD	45	M/F	71	5000 IU D3/d & 320 mg Ca/d	1 yr	4% inc. lumbar spine & 23.4% inc. hip BMD
CT, controlled trial; SAD, single arm den; OSD, open study design; F, female; M, male; D3, cholecalciferol; D2, ergocalciferol; Ca, calcium;/d, daily; wkly, weekly; mths, months; Inc., increase yr, year; yrs, years *added calcium supplements to ensure intakes were 1200-1500 mg/d							

**Table 5:** Intervention with vitamin D analogues (with / without calcium) on BMD.

Study	Study design	n	Sex	Mean age (years)	Treatment	Length	Results
**Vitamin D analogues**							
Sato *et al*. 1999 (70)	CT	86	M/F	70	40 IU Ac/d	2 yrs	Decreased loss of BMD compared to placebo
Ebeling *et al*. 2001 (72)	CT	41	M	57.5	20 IU Cl/d	2 yrs	No effect
Gallagher *et al*. 2001 (73)	CT	489	F	72	20 IU Cl/d	2 yrs	Increase in spine BMD
**Vitamin D analogues with calcium**							
Sambrook *et al*. 1993 (111)	CT	103	M/F	46	24 IU Cl & 1000 mg Ca/d	1 yr	Reduced corticosteroid bone loss in the lumbar spine.
Orimo *et al*. 1994 (98)	CT	80	F	70	40 IU Ac & 300 mg Ca/d	1 yr	Increase in lumbar spine (L2-L4) BMD in 0.65%
Shikari *et al*. 1996 (99)	CT	113	F	70	30 IU Ac & 300 mg Ca/d	2 yrs	Increase in lumbar spine (L2-L4) BMD (1.81-2.32%)
Sato *et al*. 1997 (116)	CT	64	M/F	68	40 IU Cl & 300 mg Ca/d	6 mths	Improvement in BMD on intact side of stroke subjects
Lambrinoudaki *et al*. 1999 (118)	CT	81	F	31	20 IU Cl & 1200 mg Ca/d	2 yrs	Increase in BMD at lumbar spine
Stempfle *et al*. 1999 (86)	CT	132	M/F	50	10 IU Cl & 1000 mg Ca/d	3 yrs	No effect
Sambrook *et al.* 2000 (119)	CT	65	M/F	46	20-30 IU Cc & 600 mg Ca/d	2 yrs	Reduced bone loss in proximal femur
CT, controlled trial; F, female; M, male; Cl, Calcitriol; Ac, Alfacalcidol; Ca, Calcium;/d, daily; wkly, weekly;mths, months; yr, year; yrs, years							

Therefore, though studies to date have been conflicting, vitamin D supplementation has the potential to positively benefit muscle function which could subsequently lead to a decreased propensity to fall and fracture. 

## 9. Falls and Vitamin D

Given the potential for vitamin D to modulate muscle strength, it is perhaps not surprising that a large number of studies which have investigated the effects of intervention with vitamin D alone or in combination with calcium on risk of falls [Table 6, Table 7]. Of six trials that have supplemented with vitamin D alone, two [77,143] have reported significant reductions in fall rate following supplementation with vitamin D at 1000 or 800IU of vitamin D2/d respectively. Importantly, however, seven out of nine studies [89,144,145,146,147,148,149] that supplemented with vitamin D in combination with calcium have reported significant reductions in falls. Therefore, as with fracture prevention, vitamin D appears to be more effective in reducing the risk of falls when used in combination with calcium. Significantly, one meta-analysis of 5 RCTs [150] concluded that vitamin D reduced the risk of falling by 22% while another more recent meta-analysis [151] of eight RCTs concluded that supplementation with vitamin D in the region of 700-1,000 IU/d reduced falls by 19% with doses below this level ineffective. It has been suggested that vitamin D supplementation exerts greater effects on institutionalized *versus* free-living individuals [145], a finding which may be explained by a number of factors. Individuals in care typically have lower serum concentrations of vitamin D compared to free-living individuals [152] leaving them pre-disposed to muscle weakness and increased risk of falling and also more likely to benefit from supplementation. Furthermore, compliance with the supplementations regimen may be better among institutionalised individuals where supplements are provided by caregivers. Nevertheless, not all trials of institutionalized individuals have produced significant findings [78]. 

In summary, based on the evidence reported, vitamin D, particularly when supplemented with calcium, can have a beneficial effect on the prevention of falls and thus fractures. 

**Table 6:** Intervention with vitamin D or vitamin D analogues on falls.

Study	Study design	n	Sex	Mean age (years)	Treatment	Duration	Results
Graafmans *et al*. 1996 (142)	CT	354	M/F	>70	400 IU D3/d (2 yrs)	7 mths	No effect on falls
Sato *et al*. 1999 (70)	CT	86	M/F	70	40 IU Ac/d	2 yrs	No effect on falls
Latham *et al*. 2002 (136)	CT	243	M/F	79	300,000 IU D3 once	6 mths	No effect on falls
Trivedi *et al*. 2003 (75)	CT	2686	M/F	75	100,000 IU D3 quartley	5 yrs	No effect on falls
Sato *et al*. 2005 (77)	CT	96	F	74	1,000 IU D2/d	2 yrs	59% reduction in falls and increase in size/number type 2 muscle
Law *et al*. 2006 (78)	OSD	3717	M/F	85	1,100 IU D2/d	10 mths	Noeffect
Broe *et al*. 2007 (143)	CT	124	M/F	89	800 IU D2/d	5 mths	72% lower fall rate
CT, controlled trial; F, female; M, male; D3, cholecalciferol; D2, ergocalciferol;Ac, Alfacalcidol /d, daily; wkly, weekly; Ca, calcium;							

**Table 7:** Intervention with vitamin D or vitamin D analogues (with / without calcium) on falls.

Study	Study design	n	Sex	Mean age (years)	Treatment	Duration	Results
DawsonHughes *et al.* 1997 (84)	CT	389	M/F	>65	700 IU D3/ 500 mg Ca/d	3 yrs	No effect on falls
Pfeifer *et al*. 2000(144)	CT	148	F	70-86	800 IU D3/1,200 mg Ca/d	2 mths	Reduced body sway (9%) and 1 yr follow up lower number of falls
Chapuy *et al*. 2002 (87)	CT	583	F	85	800IU D3/1200 mg Ca/d	2 yrs	No effects on falls
Larsen *et al*. 2002 (89)	IT	5771	F	74	400 IU D3/1000 mg Ca/d	3.5yrs	Reduced risk of severe falling by 12%
Bischoff *et al.* 2003 (145)	CT	122	F	>65	800IU D3/1200 mg Ca/d	12 wks	49% reduction in falling and improved muscoskeletal function
Dukas *et al*. 2004(146)	CT	378	M/F	75	40 IU Ac/d	9 mths	Reduction in falls only with Ca intake >512 mg/d in addition
Flicker *et al.* 2005 (147)	CT	625	M/F	83	10,000 IU D2/wkly*	2 yrs	Reduction in incidence ratio for falls
Bischoff *et al.* 2006 (148)	CT	445	M/F	>65	700 IU D3/500 mg Ca/d	3 yrs	46% reduction falls in womem (65% reduction in non-active women)
Pfeifer *et al*. 2009 (149)	CT	242	M/F	>70	800 IU D3/1000mg Ca/d	18 mths	27% reduction in falls & 28% decrease in body sway
CT, controlled trial; F, female; M, male; D3, cholecalciferol; D2, ergocalciferol;Ac, Alfacalcidol /d, daily; wkly, weekly; Ca, calcium;*changed to 1000 IU D2 daily with 600 mg calcium							

## 10. Bone Health, Vitamin D and Inflammation

Increasing age is not only associated with a decrease in BMD and muscle strength but is also associated with marked changes in immune and inflammatory responses. Studies conducted predominantly in individuals >70 y of age have demonstrated that, while the most striking age-related changes have been observed in the cell-mediated arm of the immune system, age-associated restructuring of the immune system is a consequence of alterations in virtually all of its components.These alterations result in the up-regulation of some components of the immune system as well as diminished function of others [153,154]. Changes include decreased mature lymphocyte function, decreased replication of hematopoietic cells [153] and an up-regulation in the production of pro-inflammatory cytokines such as interleukin 6 [IL-6] and tumour necrosis factor-alpha [TNF-α] [154]. 

Inflammation is considered a characteristic of osteoporosis [155] and research has focused on the effects of cytokines on bone metabolism [156]. The cytokines IL-1, IL-6 and TNF-α have been shown to regulate bone [157] and evidence from animal models has suggested that these cytokines are associated with the development of osteoporosis [154,155] through stimulation of osteoclastogenesis and subsequent bone resorption [158]. *In vitro* studies have indicated that TNF-α and IL-1 can stimulate human osteoclastic bone resorption [159], possibly owing to increased RANKL expression in a synergistic relationship; with TNF-α depending on the presence of IL-1 for optimal osteoclast formation [160]. 

To date, of the pro-inflammatory markers related to bone, the most studied is IL-6, the production of which can be stimulated by PTH [161]. However, findings from *in vitro* studies have been inconsistent, with reports that IL-6 stimulates both bone formation [162] and resorption [163]. Human studies have also produced inconsistent results on the relationship between IL-6 and bone health. A recent longitudinal study [164], reported that baseline IL-6 and change in IL-6 over a period of 2.9 years were consistently associated with bone loss in elderly adults. These findings are supported by a similar study in post-menopausal women [165]. Two other cross-sectional studies found no significant association between IL-6 and BMD [166,167] and another longitudinal study reported a positive association between IL-6 and bone loss in the lumbar spine [168]. The associations between other cytokines and bone health have also been assessed; TNF-α is associated with bone resorption [169], as is IL-17, which is reported to act synergistically with TNF-α to promote bone turnover in rheumatoid arthritis patients [170]. Conversely, some animal studies have suggested that IL-17 is protective in oestrogen-derived bone loss which commonly occurs during the menopause [171]. 

The relationship between polymorphisms in cytokine genes, which alter expression of the particular cytokine, and bone health has been investigated with varying results. Polymorphisms in the IL-1 receptor antagonist [IL-1ra] gene in post-menopausal women have been associated with lower BMD and increased fracture risk [172,173]; however, such associations with this polymorphism are not evident in all trials [174]. Other polymorphisms that have been identified include the 174 GG polymorphism in IL-6; this polymorphism results in a higher expression of IL-6 protein and has been associated with increased bone resorption [175]. This particular polymorphism has also been reported as a potential risk factor for hip fracture among late postmenopausal women without oestrogen replacement therapy and those with inadequate calcium intakes [176]. Studies have also indicated that polymorphisms in the TNFRSF1B gene, which codes for a TNF receptor, also contain polymorphisms which show associations with BMD [177,178]. This relationship between bone and cytokines is further strengthened by another study which reported that a polymorphism in transforming growth factor [TGF]-β1 is associated with increased bone mass and is less common in individuals with osteoporotic fractures [173].

Evidence from both epidemiological and observational studies has highlighted the immunoregulatory effects of vitamin D; research suggesting a potential role for the vitamin in the management of immunological disorders such as multiple sclerosis [179] and rheumatoid arthritis [180]. The VDR are located in of a variety of immune cells, including monocytes and macrophages [181]. Following binding of 1,25 [OH]_2_D to the VDR, monocyte and macrophage activity is up regulated enhancing the host defences and an immunosuppressive effect is observed in lymphocytes decreasing the activity of T and B cells [182]. A number of studies have reported that vitamin D, in the form of 1,25[OH]_2_D3, can down regulate cytokine production. For example, *in vitro* studies have reported a significant decrease in IL-6 and TNF-α with vitamin D [183,184]. *In vivo* studies have also reported positive associations. For example, Zhu *et al* [185] investigated an animal model of inflammatory bowel disease and reported that vitamin D, in the form of 1,25[OH]_2_D, downgraded the production of TNF-α. This finding is supported by other studies investigating clinical populations which have reported positive effects of vitamin D supplementation on cytokine profiles in patients with congestive heart failure [186] and multiple sclerosis [187]. Moreover, in relation to bone health, one study has investigated the potential of vitamin D to alter cytokine production in individuals at increased risk of fracture and concluded that 20 IU/day of calcitriol for 6 months decreased both IL-1 and TNF-α concentrations and increased BMD in post-menopausal women with osteoporosis [188].

In summary, while not assessed in the majority of vitamin D intervention studies to date, it is possible given the immunoregulatory effects of vitamin D together with the reported inflammatory aetiology of osteoporosis, that the beneficial effects of vitamin D on fracture risk may be mediated, at least in part, by an effect of vitamin D on cytokine concentration. Clearly there is a need for more studies, both observational and intervention studies, to determine whether vitamin D status favourably modulates bone health through an effect on the immune and inflammatory systems.

## 11. Conclusions

It is clear that vitamin D is essential for bone health; with insufficient intakes resulting not only in the classical deficiency diseases of rickets and osteomalacia but also in increased bone metabolism and enhanced fracture risk. With evidence accumulating of inadequate vitamin D status in many countries worldwide and particularly in older people, who represent an ever increasing section of the population, maintaining bone health and decreasing fracture risk is set to become an even greater economic and social challenge over the coming decades. The evidence from research findings to-date indicates that supplementation with vitamin D in those most at-risk of impaired bone health (immobile or institutionalized elderly) has a beneficial effect on fracture prevention. Research clearly suggests vitamin D not only improves BMD but also enhances muscle function leading to a decreased number of falls and has the potential to modulate the effect of pro-inflammatory cytokines on bone metabolism. The level of supplementation required for an optimal effect on fracture prevention, however, is still under debate with multiple studies indicating different dosage regimens. The majority of trials and meta-analyses indicate that a dose of vitamin D of 800 IU/d in combination with a sufficient intake of calcium is optimal, albeit some studies suggest an even greater benefit at higher intakes. Further studies are required to confirm the optimal dose of vitamin D required to reduce fracture risk in older people. In addition, further research is warranted in order to explore the emerging and potentially exciting effect of vitamin D on pro-inflammatory cytokines and bone health. 

## References

[1] Holick M.F. (2004). Sunlight and vitamin D for bone health and prevention of autoimmune diseases cancers, and cardiovascular disease. Am. J. Clin. Nut..

[2] Diamond T.H., Eisman J.A., Mason R.S., Nowson C.A., Pasco J.A., Sambrook P.N, Wark J.D. (2005). Vitamin D and adult bone health in Australia and New Zealand:a position statement. MJA.

[3] Holick M.F. (1994). McCollum Award Lecture, Vitamin D: New Horizons for the 21st Century. Am. J. Clin. Nut..

[4] Holick M.F. (2003). Vitamin D: a millennium perspective. J. Cell. Biochem..

[5] Chen T. (2007). Factors that affect the cutaneous synthesis and dietary sources of vitamin D. Arch. Biochem. Biophysics.

[6] Matsuoka L.Y., Ide L., Wortsman J., MacLaughlin J.A., Holick M.F. (1987). Sunscreens suppress vitamin D3 synthesis. J. Clin. Endocrinol. Metab..

[7] Holick M.F. (1995). Environmental factors that influence the cutaneous production of vitamin D. Am. J. Clin. Nutr..

[8] Josefsson W., Landelius T. (2000). Effects of clouds on UV irradiance as estimated from cloud amount, cloud type, precipitation, global radiation and sunshine duration. J. Geo. Research..

[9] Agarwal K.S., Mughal M.Z., Upadhyay P., Berry J.L., Mawer E.B., Puliyel J.M. (2002). The impact of atmospheric pollution on vitamin D status of infants and toddlers in Delhi, India. Arch. Dis. Child..

[10] Matsuoka L.Y., Wortsman J., Haddad J.G., Kolm P., Hollis B.W. (1991). Racial pigmentation and the cutaneous synthesis of vitamin D. Arch. Dermatol..

[11] MacLaughlin J., Holick M.F. (1985). Aging decreases the capacity of human skin to produce vitamin D3. J. Clin. Invest..

[12] Holick M.F., Biancuzzo R.M., Chen T.C., Klein E.K., Young A., Douglass B., Reitz R., Salameh W., Ameri A., Tannenbaum A.D. (2008). Vitamin D2 is as effective as Vitamin D3 in maintaining circulating concentrations of 25-Hydroxyvitamin D status. J. Clin. Endocrinol. Metab..

[13] Trang H.M., Cole D.E., Rubin L.A., Pierratos A., Siu S., Vieth R. (1998). Evidence that vitamin D3 increases serum 25-hydroxyvitamin D more efficiently than does vitamin D2. Am. J. Clin. Nut..

[14] Armas L.A., Hollis B.W., Heaney R.P. (2004). Vitamin D2 is much less effective than vitamin D3 in humans. J. Clin. Endocrinol. Metab..

[15] Hill T.R., O’Brien M.M., Cashman K.D., Flynn A., Kiely M. (2004). Vitamin D intakes in 18-64 year old Irish adults. Eur. J. Clin. Nut..

[16] British Paediatric Association (1956). Hypercalcaemia in infants and vitamin D. BMJ.

[17] Chen T.C., Heath H., Holick M.F. (1993). An update on the vitamin D content of fortified milk from the United States and Canada. N. Engl. J. Med..

[18] Calvo M.S., Whiting S.J., Barton C.N. (2004). Vitamin D fortification in the United States and Canada: current status and data needs. Am. J. Clin. Nutr..

[19] Bikle D. (2007). Vitamin D Insufficiency/Deficiency in Gastrointestinal Disorders. J. Bone. Min. Res..

[20] Avioii L.V. (1969). Absorption and metabolism of vitamin D3 in man. Am. J. Clin. Nutr..

[21] Driscoll R.H., Meredith S.C., Sitrin M., Rosenberg I.H. (1982). Vitamin D deficiency and bone disease in patients with Crohns disease. Gastroenterology.

[22] Sitrin M.D., Pollack K.L., Bolt M.G., Rosenberg I.H. (1982). Comparison of vitamin D and 25-hydroxyvitamin D absorption in the rat. Am. J. Physiol..

[23] Bouillon R., De Groot L., Jameson J.L., Burger H.G. (2001). Vitamin D: from photosynthesis, metabolism, and action to clinical applications. Endocrinology.

[24] Holick M.F. (2006). Resurrection of vitamin D deficiency and rickets. J. Clin. Invest..

[25] Khosla S. (2001). The OPG/RANKL/RANK system. Endocrinology.

[26] Zittermann A., Schleithoff S.S., Tenderich G., Berthold H.K., Korfre R., Stehle P. (2003). Low vitamin D status: a contributing factorin the pathogenesis of congestive heart failure?. J. Am. Coll. Cardiol..

[27] Holick M.F. (2007). Vitamin D deficiency. N. Engl. J. Med..

[28] Food Safety Authority of Ireland (1999). [1999]: Recommended Dietary Allowances for Ireland.

[29] (1997). Dietary Reference Values for Food Energy and Nutrients for the United Kingdom. Report 41. Report of the Panel on Dietary Reference Values of the Committee on Medical Aspects of Food Policy.

[30] Food and Nutrition Board Institute of Medicine. (1997). Dietary reference intakes for Calcium, Magnesium, Phosphorus, Vitamin D and Fluoride.

[31] FAO/World Health Organization (1996). Human vitamin and mineral requirements: a report of the joint FAO/WHO expert consultation, Bangkok, Thailand.

[32] UK Department of Health (1998). Nutrition and bone health: with particular reference to calcium and vitamin D. Report on Health and Social subjects.

[33] McKenna M., Freaney R., Meade A., Muldowney F. (1985). Hypovitaminosis D and elevated serum alkaline phosphatase in elderly Irish people. Am. J. Clin. Nutr..

[34] McKenna M., Freaney R. (1998). Secondary hyperparathyroidism in the elderly: means to defining hypovitaminosis D. Osteoporos. Int..

[35] Zittermann A., Scheld K., Stehle P. (1998). Seasonal variations in vitamin D status and calcium absorption do not influence bone turnover in young women. Eur. J. Clin. Nutr..

[36] Van der Wielen R.P., Lowik M.R., Van den Berg H., De Groot LC., Haller J., Moreiras O., van Staveren W.A. (1995). Serum vitamin D concentrations among elderly people in Europe. Lancet.

[37] McKenna M. (1992). Differences in vitamin D status between countries in young adults and elderly. Am. J. Med..

[38] Cashman K.D., Hill T.R., Lucey A.J., Taylor N., Seamans K.M., Muldowney S., Fitzgerald A.P., Flynn A., Barnes M.S., Horigan G. (2008). Estimation of the dietary requirement for vitamin D in healthy adults. Am. J. Clinl. Nut..

[39] Hill T.R., O’Brien M.M., Cashman K.D., Flynn A., Kiely M. (2004). Vitamin D intakes in 18-64 year old Irish adults. Eur. J. Clin. Nut..

[40] Henderson L., Irving K., Gregory J., Bates C.J., Prentice A., Perks J., Swan G., Farron M. (2003). The National Diet and Nutrition Survey: adults aged 19 to 64 years-vitamin and mineral intake and urinary analysis. Volume 3.

[41] Park Y.K., Barton C.N., Calvo M.S. (2001). Dietary contributions to serum 25 [OH] vitamin D levels [25[OH] D] differ in black and white adults in the United States: Results from NHANES III. J. Bone Miner. Res..

[42] Rasmussen L.B., Hansen G.L., Hansen E., Koch B., Mosekilde L., Mølgaard C., Sørensen O.H., Ovesen L. (2001). Vitamin D: should the supply in the Danish population be increased?. Int. J. Food Sci. Nutr..

[43] Ovesen L., Andersen R., Jakobsen J. (2003). Geographical differences in vitamin D status, with particular reference to European countries. Proc. Nutr. Soc..

[44] Van Der Wielen R.P.J., Lowik M.R.H., Van Den Berg H., De Groot L., Haller J., Moreiras O., Van Staveren W.A. (1995). Serum vitamin D concentrations among elderly people in Europe. Lancet..

[45] Barnes M.S., Robson J.P., Bonham M.P., Strain J., Wallace J. (2006). Vitamin D: Status, Supplementation and Immunodulation. Cur. Nut. Food. Sci..

[46] Vieth R., Chan P., MacFarlane G. (2001). Efficacy and safety of vitamin D3 intake exceeding the lowest observed adverse effect level. Am. J. Clin. Nutr..

[47] Todd M.A., Bailey R.R., Espiner E.A., Lynn K.L. (1987). Vitamin D2 for the treatment of chilblains-a cautionary tale. N Z. Med. J..

[48] Barrueto F. Jr., Wang-Flores H.H., Howland M.A., Hoffman R.S., Nelson L.S. (2005). Acute vitamin D intoxication in a child. Pediatrics.

[49] Vieth R., Pinto T.R., Reen B.S., Wong M.M. (2002). Vitamin D poisoning by table sugar. Lancet.

[50] Matkovic V., Jelic T., Wardlaw G.M., Ilich J.Z., Goel P.K., Wright J.K., Andon M.B., Smith K.T., Heaney R.P. (1994). Timing of peak bone mass in Caucasian females and its implication for the prevention of osteoporosis. J. Clin. Invest..

[51] Heaney R.P., Abrams S., Dawson-Hughes B., Looker A., Marcus R., Matkovic V., Weaver C. (2000). Peak bone mass Osteoporos. Int..

[52] Seeman E., Marcus R., Feldman D., Kelsey J. (1996). The effects of tobacco and alcohol use on bone. Osteoporosis.

[53] Emaus N., Berntsen G.K., Joakimsen R.M., V Fønnebø. (2005). Longitudinal changes in forearm bone mineral density in women and men aged 25-44 years: the Tromsø Study: a population-based study. Am. J. Epidemiol..

[54] Weitzmann N.M., Pacifici R. (2006). Estrogen deficiency and bone loss: an inflammatory tale. J. Clin. Invest..

[55] Slemenda C.W., Hui S.L., Longcope C., Wellman H., Johnston C.C. (1990). Predictors of bone mass in perimenopausal women. Ann. Intern. Med..

[56] Lips P. (2001). Vitamin D deficiency and secondary hyperparathyroidism in the elderly: consequences for bone loss and fractures and therapeutic implications. Endocrinology. Rev..

[57] World Health Organisation (1994). Assessment of fracture risk and its application to screening for postmenopausal osteoporosis. Technical Report Series 843..

[58] Binkley N.C., Schmeer P., Wasnich R.D., Lenchik L. (2002). What are the criteria by which a densitometric diagnosis of osteoporosis can be made in males and non-Caucasians?. J. Clin. Densitom..

[59] Cummings S.R., Black D.M., Nevitt M.C., Browner W., Cauley J., Ensrud K., Genant H.K., Palermo L., Scott J., Vogt T.M. (1993). Bone density at various sites for prediction of hip fractures. The Study of Osteoporotic Fractures Research Group. Lancet.

[60] Marshall D., Johnell O., Wedel H. (1996). Meta-analysis of how well measures of bone mineral density predict occurrence of osteoporotic fractures. BMJ.

[61] Delmas P.D., Marin F., Marcus R., Misurski D.A., Mitlak B.H. (2007). Beyond Hip: Importance of Other Nonspinal Fractures. Am. J. Med..

[62] Haentjens P., Autier P., Barette M., Boonen S. (2001). The economic cost of hip fractures among elderly women. A one year prospective, observational cohort study with matched pair analysis. Belgian Hip Fracture Study Group. J. Bone. Joint. Surg. Am. Ed..

[63] Cooper C. (1997). The crippling consequences of fractures and their impact on quality of life. Am. J. Med..

[64] Cauley J.A., Thompson D.E., Ensrud K.C., Scott J.C., Black D. (2000). Risk of mortality following clinical fractures. Osteo.Intern..

[65] Melton L.J. (1993). Hip fractures: a worldwide problem today and tomorrow. Bone.

[66] Heikinheimo R.J., Inkovaara J.A., Harju E.J., Haavisto M.V., Kaarela R.H., Kataja J.M., Kokko A.L., Kolho L.A., Rajala S.A. (1992). Annual injection of vitamin D and fractures of aged bones. Calcified. Tissue. Int..

[67] Hayashi Y., Fujita T., Inoue T. (1992). Decrease of vertebral fracture in osteoporosis by administration of 1a-hydroxyvitamin D3. J. Bone. Min. Res..

[68] Tilyard M.W., Spears G.F.S., Thomson J., Dovey S. (1992). Treatment of postmenopausal osteoporosis with calcitriol and calcium. N. Engl. J. Med..

[69] Lips P., Graafmans W.C., Ooms M.E., Bezemer P.D., Bouter L.M. (1996). Vitamin D supplementation and fracture incidence in elderly persons. Ann. Intern. Med..

[70] Sato Y., Manabe S., Kuno H., Oizumi K. (1999). Amelioration of osteopenia and hypovitaminosis D by 1a-hydroxyvitamin D3 in elderly patients with Parkinson’s disease. J. Neurosurg. Psychiatry.

[71] Peacock M., Liu G., Carey M., McClintock R., Ambrosius W., Hui S., Johnston C.C. (2000). Effect of calcium or 25OH vitamin D3 dietary supplementation on bone loss at the hip in men and women over the age of 60. J. Clin. Endocrinol. Metab..

[72] Ebeling P.R., Wark J.D., Yeung S., Poon C., Salehi N., Nicholson G.C., Kotowicz M.A. (2001). Effects of calcitriol or calcium on bone mineral density, bone turnover, and fractures in men with primary osteoporosis: a two-years randomized, double blind, double placebo study. J. Clin. Endocr. Metab..

[73] Gallagher J.C., Fowler S.E., Detter J.R., Sherman S.S. (2001). Combination treatment with estrogen and calcitriol in the prevention of age-related bone loss. J. Clin. Endocrinol. Metab..

[74] Meyer H.E., Smedshaug G.B., Kvaavik E., Falch J.A., Tverdal A., Pedersen J.I. (2002). Can vitamin D supplementation reduce the risk of fracture in the elderly? A randomized controlled trial. J. Bone. Miner. Res..

[75] Trivedi D.P., Doll R., Khaw K.T. (2003). Effect of four monthly oral vitamin D3 [cholecalciferol] supplementation on fractures and mortality in men and women living in the community: randomised double blind controlled trial. BMJ.

[76] Grant A.M., Avenell A., Campbell M.K., Cooper C., Donaldson C., Francis R.M., Gillespie W.J., Robinson C.M., Torgerson D.J. (2005). Oral vitamin D3 and calcium for secondary prevention of low trauma fractures in elderly people (Randomised Evaluation of Calcium Or Vitamin D, RECORD): a randomized placebo-controlled trial. Lancet.

[77] Sato Y., Iwamoto J., Kanoko T., Satoh K. (2005). Low-Dose Vitamin D Prevents Muscular Atrophy and Reduces Falls and Hip Fractures in Women after Stroke: A Randomized Controlled Trial. Cerebrovasc. Dis..

[78] Law M., Withers H., Morris J., Anderson F. (2006). Vitamin D supplementation and the prevention of fractures and falls: results of a randomised trial in elderly people in residential accommodation. Age Ageing.

[79] Lyons R.A., Johansen A., Brophy S., Newcombe R., Phillips C., Lervy B., Evans R., Wareham K., Stone M. (2007). Preventing fractures among older people living in institutional care: a pragmatic randomised double blind placebo controlled trial of vitamin D supplementation. Osteoporos. Int..

[80] Smith H., Anderson F., Raphael H., Maslin P., Crozier S., Cooper C. (2007). Effect of annual intramuscular vitamin D on fracture risk in elderly men and women-a population-based, randomized, double-blind, placebo-controlled trial. Rheumatology.

[81] Chapuy M.C., Arlot M.E., Duboeuf F., Brun J., Crouzet B., Arnaud S., Delmas P.D., Meunier P.J. (1992). Vitamin D3 and calcium to prevent hip fractures in elderly women. N. Engl. J. Med..

[82] Orimo H., Shiraki M., Hayashi Y., Hoshino T., Onaya T. (1994). Effects of 1a-hydroxyvitamin D3 on lumbar bone mineral density and vertebral fractures in patients with postmenopausal osteoporosis. Calcif. Tissue. Int..

[83] Shiraki M., Kushida K., Yamazaki K., Nagai T., Inoue T., Orimo H. (1996). Effects of 2 year’s treatment of osteoporosis with 1a-hydroxy vitamin D3 on bone mineral density and incidence of fracture: a placebo-controlled, double blind prospective study. Endocr. J..

[84] Dawson-Hughes B., Harris S.S., Krall E.A., Dallal G.E. (1997). Effect of calcium and vitamin D supplementation on bone density in men and women 65 years of age or older. N. Engl. J. Med..

[85] Komulainen M., Kroger H., Tuppurainen M.T., Heikkinen A.M., Alhava E., Honkanen R., Saarikoski S. (1998). HRT and vitamin D in prevention of non-vertebral fractures in postmenopausal women; a 5 year randomized trial. Maturitas.

[86] Stempfle H.U., Werner C., Echtler S., Wehr U., Rambeck W.A., Siebert U., Üderfuhr P., Angermann C.E., Theisen K., Gärtner R. (1999). Prevention of osteoporosis after cardiac transplantation. Transplantation..

[87] Chapuy M.C., Pamphile R., Paris E., Kempf C., Schlichting M., Arnaud S., Garnero P., Meunier P.J. (2002). Combined calcium and vitamin D3 supplementation in elderly women: confirmation of reversal of secondary hyperparathyroidism and hip fracture risk: the Decalyos II Study. Osteoporos. Int..

[88] Harwood R.H., Sahota O., Gaynor K., Masud T., Hosking D.J. Nottingham Neck of Femur [NONOF] Study.  (2004). A randomised, controlled comparison of different calcium and vitamin D supplementation regimens in elderly women after hip fracture: the Nottingham Neck of Femur [NONOF] Study. Age. Ageing..

[89] Larsen E.R., Mosekilde L., Foldspang A. (2004). Vitamin D and calcium supplementation prevents osteoporotic fractures in elderly community dwelling residents: a pragmatic population-based 3-year intervention study. J. Bone. Miner. Res..

[90] Flicker L., MacInnis R.J., Stein M.S., Scherer S.C., Mead K.E., Nowson C.A., Thomas J., Lowndes C., Hopper J.L., Wark J.D. (2005). Should older people in residential care receive vitamin D to prevent falls? Results of a randomized trial. J.Am. Geriatr. Soc..

[91] Porthouse J., Cockayne S., King C., Saxon L., Steele E., Aspray T., Baverstock M.;Birks, Dumville J., Francis R., Iglesias C., Puffer S., Sutcliffe A., Watt I, Torgerson D.J.  (2005). Randomised controlled trial of calcium and supplementation with cholecalciferol [vitamin D3] for prevention of fractures in primary care. BMJ.

[92] Jackson R.D., LaCroix A.Z., Gass M., Wallace R.B., Robbins J., Bassford T., Berresford S., Black H., Blanchette P., Bonds D. (2006). Calcium plus vitamin D supplementation and the risk of fractures. N. Engl. J. Med..

[93] Pfeifer M., Begerow B., Minne H.W., Suppan K., Fahrleitner-Pammer A., Dobnig H. (2009). Effects of a long-term vitamin D and calcium supplementation on falls and parameters of muscle function in community-dwelling older individuals. Osteoporos. Int..

[94] Chapuy M.C., Arlot M.E., Delmas P.D., Meunier P.J. (1994). Effect of calcium and cholecalciferol treatment for three years on hip fractures in elderly women. BMJ.

[95] Avenell A., Gillespie W.J., Gillespie L.D., O’Connell D. (2009). Vitamin D and vitamin D analoques for preventing fractures associated with involutional and postmenopausalosteoporosis. Cochrane Database Syst. Rev..

[96] Tang B.M.P., Eslick G.D., Nowson C., Smith C., Bensoussan A. (2007). Use of calcium or calcium in combination with vitamin D supplementation to prevent fractures and bone loss in people aged 50 years and older: a meta-analysis. Lancet.

[97] Bischoff-Ferrari H.A., Willett W.C., Wong J.B., Giovannucci E., Dietrich T., Dawson-Hughes B. (2005). Fracture prevention with vitamin D supplementation: a meta-analysis of randomized controlled trials. JAMA.

[98] Boonen S., Lips P., Bouillon R., Bischoff-Ferrari H.A, Vanderschueren D., Haentjens P.  (92). Need for additional calcium to reduce the risk of hip fracture with vitamin D supplementation: Evidence from a comparative meta-analysis of randomized controlled trials. J. Clin. Endocrinol. Metabol..

[99] Bischoff-Ferrari H.A., Willett W.C., Wong J.B., Stuck A.E., Staehelin H.B., Orav E.J., Thoma A., Kiel D.P., Henschkowski J. (2009). Prevention of non-vertebral fractures with oral vitamin D and dose dependency. A meta-analysis of randomized controlled trials. Arch. Intern. Med..

[100] Avernell A., Gillespie W.J., Gillespie L.D., O'Connell D. (2005). Vitamin D and vitamin D analogues for preventing fractures associated with involutional and post-menopausal osteoporosis. Cochrane Database Syst. Rev..

[101] The DIPART (vitamin D Individual Patient Analysis of Randomized Trials) Group (2010). Patient level pooled analysis of 68 500 patients from seven major vitamin D fracture trials in US and Europe. B.M.J..

[102] Richy F., Schacht E., Bruyere O., Ethgen O., Gourlay M., Reginster J.Y. (2005). Vitamin D Analogs Versus Native Vitamin D in Preventing Bone Loss and Osteoporosis-Related Fractures: A Comparative Meta-analysis. Calcif. Tissue. Int..

[103] Khaw K.T., Sneyd M.J., Compston J. (1992). Bone density, parathyroid hormone and 25-hydroxyvitamin D concentrations in middle-aged women. Br. Med. J..

[104] Bischoff-Ferrari H.A, Dietrich T., Orav E.J., Dawson-Hughes B.  (2004). Positive association between 25-hydroxy vitamin D levels and bone mineral density: a population-based study of younger and older adults. Am. J. Med..

[105] Nordin B.E.C., Baker M.R., Horsman A., Peacock M. (1985). A prospective trial of the effect of vitamin D supplementation on metacarpal bone loss in elderly women. Am. J. Clin. Nutr..

[106] Ooms M.E., Roos J.C., Bezemer P.D., Van Der Vijgh J.F., Bouter L.M., Lips P. (1995). Prevention of bone loss by vitamin D supplementation in elderly women: a randomized double-blind trial. J. Clin. Endocrinol. Metab..

[107] Adams J.S., Kantorovich V., Wu C., Javanbakht M., Hollis B.W. (1999). Resolution of vitamin D insufficiency in osteopenic patients results in rapid recovery of bone mineral density. J. Clin. Endocrinol. Metab..

[108] Hunter D., Major P., Arden N., Swaminathan R., Andrew T., MacGregor A.J., Keen R., Snieder H., Spector T.D. (2000). A randomized controlled trial of vitamin D supplementation on preventing postmenopausal bone loss and modifying bone metabolism using identical twin pairs. J. Bone. Miner. Res..

[109] Patel R., Collins D., Bullock S., Swaminathan R., Blake G.M., Fogelman I. (2001). The effect of season and vitamin D supplementation on bone mineral density in healthy women: A double-masked crossover study. Osteoporos. Int..

[110] Dawson-Hughes B., Dallal G.E., Krall E.A., Harris S., Sokoll L.J., Falconer G. (1991). Effect of vitamin D supplementationon wintertime and overall bone loss in healthy postmenopausal women. Ann. Intern. Med..

[111] Sambrook P., Birmingham J., Kelly P., Kempler S., Nguyen T., Pocock N., Eisman J.  (1993). Prevention of corticosteroid osteoporosis. A comparison of calcium, calcitriol, and calcitonin. N. Engl. J. Med..

[112] Dawson Hughes B., Harris S.S., Krall E.L., Dallal G.E., Falconer G., Green C.L. (1995). Rates of bone loss in postmenopausal women randomly assigned to one of two dosages of vitamin D. Am. J. Clin. Nutr..

[113] Adachi J.D., Bensen W.G., Bianchi F., Cividino A., Pillersdorf S., Sebaldt R.J., Tugwell P., Gordon M., Steele M., Webber C., Goldsmith C.H. (1996). Vitamin D and calcium in the prevention of corticosteroid induced osteoporosis: a 3 year followup. J. Rheum..

[114] Bernstein C.N., Seeger L.L., Anton P.A., Artinian L., Geffrey S., Goodman W., Belin T.R., Shanahan F. (1996). A randomized, placebo-controlled trial of calcium supplementation for decreased bone density in corticosteroid-using patients with inflammatory bowel disease: a pilot study. Aliment. Pharmacol. Ther..

[115] Buckley L.M., Leib E.S., Cartularo K.S., Vacek P.M., Cooper S.M. (1996). Calcium and vitamin D3 supplementation prevents bone loss in the spine secondary to low-dose corticosteroids in patients with rheumatoid arthritis. Ann. Intern. Med..

[116] Sato Y., Maruoka H., Oizumi K. (1997). Amelioration of hemiplegia-associated osteopenia more than 4 years after stroke by 1a-hydroxyvitamin D3 and calcium supplementation. Stroke.

[117] Baeksgaard L., Andersen K.P., Hyldstrup L. (1998). Calcium and vitamin D supplementation increases spinal BMD in healthy, postmenopausal women. Osteoporos. Int..

[118] Lambrinoudaki I., Chan D.T., Lau C.S., Wong R.W., Yeung S.S., Kung A.W. (2000). Effect of calcitriol on bone mineral density in premenopausal Chinese women taking chronic steroid therapy. A randomized, double-blind, placebo controlled study. J. Rheumatol..

[119] Sambrook P., Henderson N.K., Keogh A., Maodonald P., Glanville A. (2000). Effect of calcitriol on bone loss after cardiac or lung transplantation. J. Bone. Min. Res..

[120] Cooper L., Clifton-Bligh P., Nery M.L., Figtree G., Twigg S., Hibbert E., Robinson B.G. (2003). Vitamin D supplementation and bone mineral density in early postmenopausal women. Am. J. Clin. Nutr..

[121] Meier C., Woitge H.W., Witte K., Lemmer B., Seibel M.J. (2004). Supplementation with oral vitamin D3 and calciumduring winter season prevents seasonal bone loss: a randomized controlled open-label prospective trial. J. Bone. Min. Res..

[122] Aloia J.F., Talwar S.A., Pollack S., Yeh J.  (2005). A randomized controlled trial of vitamin D3 supplementation in African American women. Arch. Inter. Med..

[123] Mocanu V., Stitt P.A., Costan A.R., Voroniuc O., Zbranca E., Luca V., Vieth R. (2009). Long-term effects of giving nursing home residents bread fortified with 125 µg [5000 IU] vitamin D3 per daily serving. Am. J. Clin. Nutr..

[124] Nelson D.A., Barondess D.A., Hendrix S.L., Beck T.J. (2000). Cross-sectional geometry, bone strength, and bone mass in the proximal femur in black and white postmenopausal women. J. Bone. Miner. Res..

[125] Aloia J.F., Mikhail M., Pagan C.D., Arunachalam A., Yeh J.K., Flaster E. (1998). Biochemical and hormonalvariables in black and white women matched for age and weight. J. Lab. Clin. Med..

[126] Gillespie L.D., Gillespie W.J., Robertson M.C., Lamb S.E., Cumming R.G., Rowe B.H. (2003). Interventions for preventing falls in elderly people. Cochrane Database Syst. Rev..

[127] Boland R. (1986). Role of vitamin D in skeletal muscle function. Endocr. Rev..

[128] Pfeifer M., Begerow B., Minne H.W., Schlotthauer T., Pospeschill M., Scholz M., Lazarescu A.D., Pollähne W. (2001). Vitamin D status, trunk muscle strength, body sway, falls, and fractures among 237 postmenopausal women with osteoporosis. Exp. Clin. Endocrinol. Diabetes..

[129] Costa E.M., Blau H.M., Feldman D. (1986). 1,25-dihydroxyvitamin D3 receptors and hormonal responses in cloned human skeletal muscle cells. Endocrinology.

[130] Simpson R.U., Thomas G.A., Arnold A.J. (1985). Identification of 1,25-dihydroxyvitamin D3 receptors and activities in muscle. J. Biol. Chem..

[131] Sorensen O.H., Lund B., Saltin B., Lund B., Andersen R.B., Hjorth L., Melsen F.;Mosekilde (1979). Myopathy in bone loss of ageing: improvement by treatment with 1 alpha-hydroxycholecalciferol and calcium. Clin. Sci. (Lond)..

[132] McComas A.J. (1996). Skeletal muscle: form and function. Champaign: Human Kinetics.

[133] Bischoff-Ferrari H.A., Dietrich T., Orav J.E., Hu F.B., Zhang Y., Karlson E.W., Dawson-Hughes B. (2004). Higher 25-hydroxy vitamin D concentrations are associated with better lower-extremity function in both active and inactive persons aged >60 yrs. Am. J. Clin. Nutr..

[134] Glerup H., Mikkelsen K., Poulsen L., Hass E., Overbeck S., Andersen H., Charles P., Eriksen E.F. (2000). Hypovitaminosis D myopathy without biochemical signs of osteomalacic bone involvement. Calcif. Tissue. Int..

[135] Grady D., Halloran B., Cummings S. (1991). 1,25-Dihydroxyvitamin D3 and muscle strength in the elderly: a randomized controlled trial. J. Clin. Endocrinol. Metab..

[136] Latham N.K., Anderson C.S., Lee A., Bennett D.A., Moseley A., Cameron I.D. (2003). A randomized, controlled trial of quadriceps resistance exercise and vitamin D in frail older people: the Frailty Interventions Trial in Elderly Subjects (FITNESS). J. Am. Geriatr. Soc..

[137] Gerdhem P., Ringsberg K.A.M., Obrant K.J., Akesson K. (2005). Association between 25-hydroxy vitamin D levels, physical activity, muscle strength and fractures in the propective population-based OPRA study of elderly women. Osteoporos. Int..

[138] Houston D.K., Cesari M., Ferruci L., Cherubini A., Maggio D., Bartali B., Johnson M.A., Schwartz G.G., Kritchevsky S.B. (2007). Association between vitamin D and physical performance: The InCHIANTI study. J. Gerontol..

[139] Garber A.J. (1983). Effects of parathyroid hormone on skeletal muscle protein and amino acid metabolism in the rat. J. Clin. Invest..

[140] Mitnick M.A., Grey A., Masiukiewicz U., Bartkiewicz M., Rios-Velez L., Friedman S., Xu L., Horowitz M.C., Insogna K. (2001). Parathyroid hormone induces hepatic production of bioactive interleukin-6 and its soluble receptor. Am. J. Physiol. Endocrinol. Metab..

[141] Han K.O., Choi J.T., Moon I.G., Jeong M.S., Yim C.H., Chung H.Y., Jang H.C., Yoon H.K., Han I.K. (2002). Nonassociation of interleukin-1 receptor antagonist genotypes with bone mineral density, bone turnover status, and estrogen responsiveness in Korean postmenopausal women. Bone.

[142] Graafmans W.C., Ooms M.E., Hofstee H.M., Bezemer P.D., Bouter L.M., Lips P. (1996). Falls in the elderly: a prospective study of risk factors and risk profiles. Am. J. Epidemiol..

[143] Broe K.E., Chen T.C., Weinberg J., Bischoff-Ferrari H.A., Holick M.F., Kiel D.P. (2007). A higher dose of vitamin D reduces the risk of falls in nursing home residents: a randomized, multiple-dose study. J. Am. Geriatr. Soc..

[144] Pfeifer M., Begerow B., Minne H.W., Abrams C., Nachtigall D., Hansen C. (2000). Effects of a short-term vitamin D and calcium supplementation on body sway and secondary hyperparathyroidism in elderly women. J. Bone. Miner. Res..

[145] Bischoff H.A., Stahelin H.B., Dick W., Akos R., Knecht M., Salis C., Nebiker M., Theiler R., Pfeifer M., Begerow B., Lew R.A., Conzelmann M. (2003). Effects of vitamin D and calcium supplementation on falls: a randomized controlled trial. J. Bone. Miner. Res..

[146] Dukas L., Bischoff H.A., Lindpaintner L.S., Schacht E., Birkner-Binder D., Damm T.N., Thalmann B., Stähelin H.B. (2004). Alfacalcidol reduces the number of fallers in a communitydwelling elderly population with a minimum calcium intake of more than 500 mg daily. J. Am. Geriatr. Soc..

[147] Flicker L., MacInnis R.J., Stein M.S., Scherer S.C., Mead K.E., Nowson C.A., Thomas J., Lowndes C., Hopper J.L., Wark J.D. (2005). Should older people in residential care receive vitamin D to prevent falls? Results of a randomized trial. J. Am. Geriatr. Soc..

[148] Bischoff-Ferrari H.A., Orav E.J., Dawson-Hughes B. (2006). Effect of cholecalciferol plus calcium on falling in ambulatory older men and women: a 3-year randomized controlled trial. Arch. Intern. Med..

[149] Pfeifer M., Begerow B., Minne H.W., Suppan K., Fahrleitner-Pammer A., Dobnig H. (2009). Effects of a long-term vitamin D and calcium supplementation on falls and parameters of muscle function in community-dwelling older individuals. Osteoporos. Int..

[150] Bischoff-Ferrari H.A, Dawson-Hughes B., Willett W.C. (2004). Effect of Vitamin D on Falls: A Meta-analysis. JAMA.

[151] Bischoff-Ferrari H.A., Dawson-Hughes B., Staehelin H.B., Orav J.E., Stuck A.E., Theiler R., Wong J., Egli A., Kiel D., Henschkowski J. (2009). Fall prevention with supplemental and active forms of vitamin D: a meta-analysis of randomised controlled trials. BMJ.

[152] Agrawal S., Krueger D., Engelke J.A., Nest L., Krause P., Drinka P., Binkley N. (2006). Vitamin D status, risedronate and bone turnover in nursing home residents. J. Am. Geriatr. Soc..

[153] Linton P.J., Dorshkind K. (2004). Age-related changesin lymphocyte development and function. Nat. Immunol..

[154] Gabay C., Kushner I. (1999). Acute-phase proteins and other systemic responses to inflammation. N. Engl. J. Med..

[155] Clowes J.A., Riggs B.L., Khosla S. (2005). The role of the immune system in the pathophysiology of osteoporosis. Immunol. Rev..

[156] Jilka R.L., Hangoc G., Girasole G., Passeri G., Williams D.C., Abrams J.S., Boyce B., Broxmeyer H., Manolagas S.C. (1992). Increased osteoclast development after oestrogen loss: mediation by interleukin-6. Science.

[157] Collin-Osdoby P., Rothe L., Anderson F. (2001). Receptor activator of NF-kappa B and osteoprotegerin expression by human microvascular endothelial cells, regulation by inflammatory cytokines, and role in human osteoclastogenesis. J. Biol. Chem..

[158] Bendixen A.C., Shevde N.K., Dienger K.M., Willson T.M., Funk C.D., Pike J.W. (2001). IL-4 inhibits osteoclast formation through a direct action on osteoclast precursors via peroxisome proliferator-activated receptor gamma 1. Proc. Natl. Acad. Sci. USA.

[159] Bertolini D.R., Nedwin G.E., Bringman T.S., Smith D.D., Mundy D.D. (1986). Stimulation of bone resorption and inhibition of bone formation *in vitro* by human tumor necrosis factor. Nature.

[160] Wei S., Kitaura H., Zhou P., Patrick R.F., Teitelbaum S.L. (2005). IL-1 mediates TNF-induced osteoclastogenesis. J. Clin. Invest..

[161] Ershler W.B., Keller E.T. (2000). Age-associated increased interleukin-6 gene expression, late-life diseases, and frailty. Annu. Rev. Med..

[162] Takeuchi Y., Watanabe S., Ishii G., Takeda S., Nakayama K., Fukumoto S., Kaneta Y., Inoue D., Matsumoto T., Harigaya K. (2002). Interleukin-11 as a stimulatory factor for bone formation prevents bone loss with advancing age in mice. J. Biol. Chem..

[163] Palmqvist P., Persson E., Conaway H.H., Lerner U.H. (2002). IL-6, leukemia inhibitory factor, and oncostatin M stimulate bone resorption and regulate the expression of receptor activator of NF-kappa B ligand, osteoprotegerin, and receptor activator of NF-kappa B in mouse calvar. J. Immunol..

[164] Ding C., Parameswaran V., Udayan R., Burgess J., Jones G. (2008). Circulating Levels of Inflammatory Markers Predict Change in Bone Mineral Density and Resorption in Older Adults: A Longitudinal Study. J. Clin. Endocrinol. Metab..

[165] Scheidt-Nave C., Bismar H., Leidig-Bruckner G., Woitge H., Seibel M.J., Ziegler R., Pfeilschifter J. (2001). Serum interleukin 6 is a major predictor of bone loss in women specific to the first decade past menopause. J. Clin. Endocrinol. Metab..

[166] McKane W.R., Khosla S., Peterson J.M., Egan K., Riggs B.L. (1994). Circulating levels of cytokines that modulate bone resorption: effects of age and menopause in women. J. Bone. Miner. Res..

[167] Kania D.M., Binkley N., Checovich M., Havighurst T., Schilling M., Ershler W.B. (1995). Elevated plasma levels of interleukin-6 in postmenopausal women do not correlate with bone density. J. Am. Geriatr. Soc..

[168] Abrahamsen B., Bonnevie-Nielsen V., Ebbesen E.N., Gram J., Beck-Nielsen H. (2000). Cytokines and bone loss in a 5-year longitudinal study-hormone replacement therapy suppresses serum soluble interleukin-6 receptor and increases interleukin-1-receptor antagonist: the Danish Osteoporosis Prevention Study. J. Bone. Miner. Res..

[169] Nanes M.S. (2003). Tumor necrosis factor- α: molecular and cellular mechanisms in skeletal pathology. Gene.

[170] Gaffen S. L. (2004). Biology of recently discovered cytokines: interleukin- 17-a unique inflammatory cytokine with roles in bone biology and arthritis. Arthritis. Res. Ther..

[171] Goswami J., Hernández-Santos N., Zuniga L.A., Gaffen S.L. (2009). A bone-protective role for IL-17 receptor signaling in ovariectomy-induced bone loss Europ. J. Immun..

[172] Kim J.G., Lim K.S., Ku S.Y., Kim S.H., Choi Y.M., Moon S.Y. (2006). Relations between interleukin-1, its receptor antagonist gene polymorphism, and bone mineral density in postmenopausal Korean women. J. Bone. Miner. Metab..

[173] Langdahl B.L., Løkke E., Carstens M., Stenkjaer L.L., Eriksen E.F. (2000). Osteoporotic fractures are associated with an 86-base pair repeat polymorphism in the interleukin-1--receptor antagonist gene but not with polymorphisms in the interleukin-1beta gene. J Bone Miner Res..

[174] Han K.O., Choi J.T., Moon I.G., Jeong M.S., Yim C.H., Chung H.Y., Jang H.C., Yoon H.K., Han I.K. (2002). Nonassociation of interleukin-1 receptor antagonist genotypes with bone mineral density, bone turnover status, and estrogen responsiveness in Korean postmenopausal women. Bone.

[175] Ferrari S.L., Ahn-Luong L., Garnero P., Humphries S.E., Greenspan S.L. (2003). Two promoter polymorphisms regulating interleukin-6 gene expression are associated with circulating levels of C-reactive protein and markers of bone resorption in postmenopausal women. J. Clin. Endocrinol. Metab..

[176] Ferrari L.S., Karasik D., Liu J., Karamohamed S., Herbert A.G., Cupples L., Kiel P.D. (2004). Interactions of Interleukin-6 Promoter Polymorphisms With Dietary and Lifestyle Factors and Their Association With Bone Mass in Men and Women From the Framingham Osteoporosis Study. J. Bone. Miner. Res..

[177] Spotila L.D., Rodriguez H., Koch M., Adams K., Caminis J., Tenenhouse H.S., Tenenhouse A. (2000). Association of a polymorphism in the TNFR2 gene with low bone mineral density. J. Bone. Miner. Res..

[178] Albagha O.M.E., Tasker P.N., McGuigan F.E.A., Reid D.M., Ralston S.H. (2002). Linkage disequilibrium between polymorphisms in the human *TNFRSF1B* gene and their association with bone mass in perimenopausal women. Hum. Mol. Genet..

[179] Hutter C., Laing P. (1996). Multiple sclerosis: sunlight, diet, immunology and aetiology. Med. Hypotheses..

[180] Merlino L.A,., Curtis J., Mikuls T.R., Cerhan J.R., Criswell L.A., Saag K.G. (2004). Vitamin D intake is inversely associated with rheumatoid arthritis: results from the Iowa Women’s Health Study. Arthritis. Rheum..

[181] Veldman C.M., Cantorna M.T., DeLuca H.F. (2000). Expression of 1,25-dihydroxyvitamin D3 receptor in the immune system. Arch. Biochem. Biophys..

[182] De Luca H.F., Cantorna M.T. (2001). Vitamin D: its role and uses in immunology. FASEB J..

[183] Muller K., Haahr P.M., Diamant M. (1992). 1,25-Dihydroxyvitamin D3 inhibits cytokine production by human blood monocytes at the post-transcriptional level. Cytokine.

[184] Evans K.N., Nguyen L., Chan J., Innes B.A., Bulmer J.N., Kilby M.D., Hewison M. (2006). Effects of 25-hydroxyvitamin D3 and 1,25-dihydroxyvitamin D3 on cytokine production by human decidual cells. Biol. Reprod..

[185] Zhu Y., Mahon B.D., Froicu M., Cantorna M.T. (2005). Calcium and 1 alpha,25-dihydroxyvitamin D3 target the TNF-alpha pathway to suppress experimental inflammatory bowel disease. Eur. J. Immunol..

[186] Schleithoff S.S., Zittermann A., Tenderich G., Berthold H.K., Stehle P., Koerfer R. (2006). Vitamin D supplementation improves cytokine profiles in patients with congestive heart failure: a double-blind, randomized, placebo-controlled trial. Am. J. Clin. Nutr..

[187] Mahon B.D., Gordon S.A., Cruz J., Cosman F., Cantorna M.T. (2003). Cytokine profile in patients with multiple sclerosis following vitamin D supplementation. J. Neuroimmunol..

[188] Inanir A., Ozoran K., Tutkak H., Mermerci B. (2004). The effects of calcitriol therapy on serum interleukin-1, interleukin-6 and tumour necrosis factor-alpha concentrations in post-menopausal patients with osteoporosis. J. Int. Med. Res..

